# Short-acting β_2_-agonist use, exacerbation risk and triple therapy in COPD: *post hoc* analyses of ETHOS

**DOI:** 10.1183/23120541.00348-2025

**Published:** 2025-12-15

**Authors:** John R. Hurst, Dave Singh, Fernando J. Martinez, Klaus F. Rabe, Patrick Darken, Niki Arya, Charlotta Movitz, Karin Bowen, Mehul Patel

**Affiliations:** 1 UCL Respiratory, University College London, London, UK; 2 Medicines Evaluation Unit, University of Manchester, Manchester University NHS Foundation Hospitals Trust, Manchester, UK; 3 Division of Pulmonary, Allergy, and Critical Care Medicine, University of Massachusetts Chan, Worcester, MA, USA; 4 LungenClinic Grosshansdorf and Christian-Albrechts University Kiel, Airway Research Center North, German Center for Lung Research (DZL), Grosshansdorf, Germany; 5 Late Respiratory and Immunology, Biometrics, Biopharmaceuticals R&D, AstraZeneca, Gaithersburg, MD, USA; 6 Late Respiratory and Immunology, Biometrics, Biopharmaceuticals R&D, AstraZeneca, Durham, NC, USA; 7 Late Respiratory and Immunology, Biometrics, Biopharmaceuticals R&D, AstraZeneca, Gothenburg, Sweden; 8 Late Respiratory and Immunology, Clinical Development, Biopharmaceuticals R&D, AstraZeneca, Cambridge, UK; 9 These authors contributed equally

## Abstract

**Background:**

Short-acting β_2_-agonist (SABA) rescue therapy can relieve COPD symptoms. We assessed post-randomisation treatment effects on exacerbations and health-related quality of life in ETHOS by rescue SABA use.

**Methods:**

In ETHOS (NCT02465567), symptomatic people with COPD and an exacerbation history were randomly assigned 1:1:1:1 to budesonide/glycopyrronium/formoterol fumarate dihydrate (BGF) (320/14.4/10 or 160/14.4/10 μg), glycopyrronium/formoterol fumarate dihydrate (GFF) (14.4/10 μg) or budesonide/formoterol fumarate dihydrate (BFF) (320/10 μg). *Post hoc* analyses assessed exacerbation rates by baseline and post-randomisation SABA use (>4 *versus* ≤4 inhalations·day^−1^), St George's Respiratory Questionnaire change from baseline by post-randomisation SABA use (>4 *versus* ≤4 inhalations·day^−1^), and post-randomisation SABA use surrounding (30 days before, day of onset, 30 days after) the first exacerbation.

**Results:**

Across treatments, higher moderate/severe exacerbation rates were observed for participants with higher (range: 1.62–2.51) *versus* lower (range: 1.14–1.51) SABA use at baseline or post-randomisation. Post-randomisation SABA use increased in the 30 days preceding, and decreased in the 30 days following, an exacerbation. Evidence of BGF benefit *versus* dual therapies in reducing moderate/severe exacerbation rates were seen regardless of SABA use level at baseline or post-randomisation, with greater BGF benefit observed *versus* GFF with higher SABA use (rate ratios (95% CI): high baseline SABA, 0.62 (0.53–0.72); high post-randomisation SABA, 0.64 (0.54–0.76)).

**Conclusion:**

These results suggest increased SABA use is associated with an impending exacerbation. Further, BGF reduces exacerbation rates regardless of SABA use, with greater benefit in those with higher SABA use.

## Shareable abstract

**Observing increased short-acting β_2_-agonist rescue inhaler use in people with COPD may facilitate early detection of an impending exacerbation and identify those more likely to benefit from additional therapeutic interventions** https://bit.ly/430HCOq

## Graphical abstract

**Figure:**
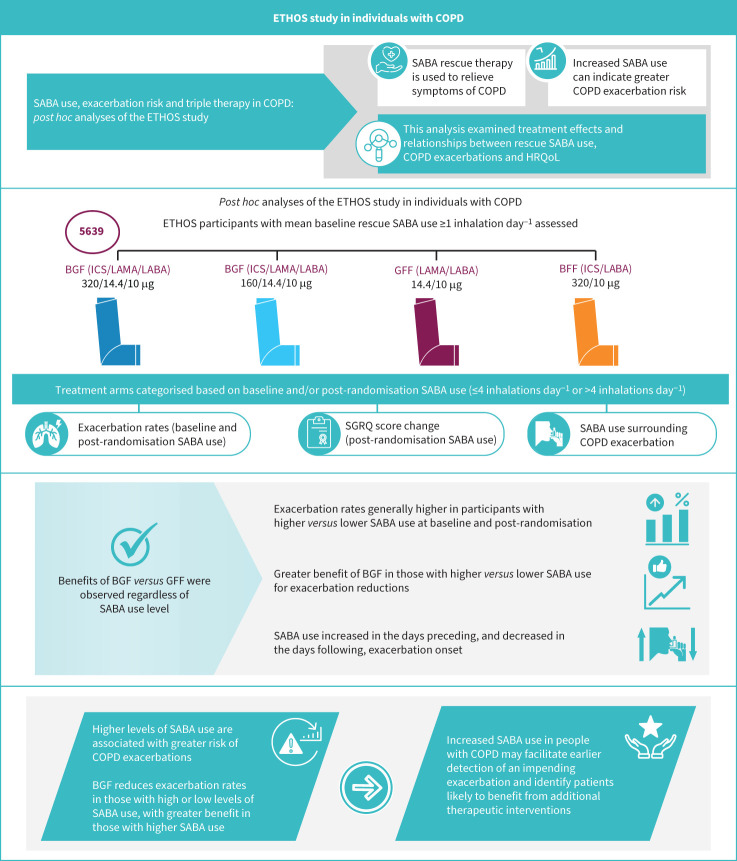
*Post hoc* analyses of the ETHOS study in individuals with COPD. SABA: short-acting β_2_-agonist; HRQoL: health-related quality of life; BGF: budesonide/glycopyrronium/formoterol fumarate dihydrate; ICS: inhaled corticosteroid; LAMA: long-acting muscarinic antagonist; LABA: long-acting β_2_-agonist; GFF: glycopyrronium/formoterol fumarate dihydrate; BFF: budesonide/formoterol fumarate dihydrate; SGRQ: St George's Respiratory Questionnaire.

## Introduction

People with COPD experience acute worsening of daily symptoms [1], which can culminate in exacerbations [2]. To relieve acute symptoms, short-acting β_2_-agonist (SABA) rescue therapy may be used [3, 4]. SABA use tends to increase with increasing COPD severity, and high SABA use can indicate poor symptom control, be associated with increased COPD exacerbation risk and reduce quality of life [3, 5–8]. In a retrospective analysis, individuals with mean SABA use of ≥2 inhalations·day^−1^ exhibited higher exacerbation rates *versus* those with mean SABA use of <2 inhalations·day^−1^ [3]. In the same study, exacerbation rates were 135% higher among those with mean SABA use of ≥10 inhalations·day^−1^ *versus* those with mean SABA use of <2 inhalations·day^−1^ [3].

In ETHOS, triple fixed-dose combination treatment with inhaled corticosteroid (ICS)/long-acting muscarinic antagonist (LAMA)/long-acting β_2_-agonist (LABA) budesonide/glycopyrronium/formoterol fumarate dihydrate (BGF) significantly reduced moderate/severe COPD exacerbation rates *versus* dual therapies (LAMA/LABA and ICS/LABA) in participants with moderate-to-very severe COPD [9].

The main aims of these analyses were to investigate: 1) whether higher SABA use is associated with a change in BGF treatment effects on exacerbation rates and health-related quality of life (HRQoL); and 2) the use of SABA in the days immediately surrounding an exacerbation. We hypothesise that: 1) rescue SABA use would increase before an exacerbation; 2) higher rescue SABA use would be associated with increased risk of exacerbations and poor HRQoL; and 3) BGF benefits would be seen irrespective of rescue SABA use level. The current *post hoc* analyses of ETHOS investigated the above, with the goal of helping clinicians recognise patients who may benefit from further intervention, including therapy escalation.

## Materials and methods

### Study design

ETHOS (NCT02465567) was a randomised, multinational, double-blind, parallel-group, Phase III study. Full study details are published elsewhere [9, 10].

At study start, participants completed a screening period (1–4 weeks; extension to 10 weeks if an exacerbation occurred). At Visit 1, existing COPD maintenance medications were adjusted for the remainder of screening: all LAMA, LABA and LAMA/LABA therapies were discontinued, participants receiving an ICS/LABA discontinued LABA use but continued to receive ICS monotherapy, with all participants receiving open-label ipratropium bromide four times daily.

At randomisation (Visit 4), participants were assigned 1:1:1:1 to one of the following treatments, twice daily over 52 weeks, *via* a single metered dose inhaler: BGF 320/14.4/10 μg, BGF 160/14.4/10 μg, glycopyrronium/formoterol fumarate dihydrate (GFF) 14.4/10 μg, or budesonide/formoterol fumarate dihydrate (BFF) 320/10 μg. Randomisation was stratified by exacerbation history (1 or ≥2 moderate or severe exacerbations in the past year), post-bronchodilator % predicted forced expiratory volume in 1 s (FEV_1_ 25%– <50% or 50%– <65%), blood eosinophil count (<150 or ≥150 cells·mm^−3^) and country.

Study protocol and informed consent forms were approved by institutional review boards (IRBs), independent ethics committees (IECs) or health authorities. Participants provided written informed consent before screening. A listing of IRBs and IECs has been published [11].

### Participants

ETHOS included symptomatic participants with moderate-to-very severe COPD and ≥1 moderate or severe COPD exacerbation in the past year (if post-bronchodilator FEV_1_ was <50% predicted normal) or ≥2 moderate exacerbations or ≥1 severe exacerbation (if FEV_1_ ≥50% predicted normal). Participants must have been taking ≥2 inhaled maintenance therapies for COPD management for ≥6 weeks before screening, which could include scheduled SABA and/or short-acting muscarinic antagonist. As-needed treatment with SABA (salbutamol sulfate) was provided as rescue medication throughout the study. SABA use was to be recorded by the participants in an electronic diary that was provided at the screening visit.

### End-points

The primary efficacy end-point in ETHOS was the annual rate of moderate/severe COPD exacerbations. Exacerbations were defined as changes in the patient's usual COPD symptoms lasting ≥2 days, beyond normal day-to-day variation and acute in onset, and warranting a regular medication change. Symptom changes must have included ≥1 major symptom (dyspnoea, sputum volume and sputum colour) and ≥1 other major symptom or minor symptom (cough, wheeze, sore throat, cold symptoms (rhinorrhea or nasal congestion), and fever without other cause). Moderate exacerbations were those leading to systemic glucocorticoid and/or antibiotic treatment for a minimum of 3 days; severe exacerbations were those resulting in hospitalisation or death. HRQoL, measured by St. George's Respiratory Questionnaire (SGRQ) total score [12], was a pre-specified secondary end-point.

### *Post hoc* analyses

As regional differences in rescue SABA use were expected, these analyses focus on settings where SABAs are commonly prescribed for COPD. Analyses were conducted in participants with mean baseline SABA use ≥1.0 inhalations·day^−1^ (rescue SABA use population) from the modified intent-to-treat (mITT) population, which included post-randomisation data from randomised and treated participants. All reported exacerbation rates are those that occurred post-randomisation.

#### Analysis 1: exacerbation rates by baseline rescue SABA use

For assessment of moderate/severe and severe exacerbation rates over 52 weeks by baseline rescue SABA use, participants were divided into those with mean SABA use of ≤4 *versus* >4 inhalations·day^−1^ over the last 7 days of screening, which was defined as “baseline” use. This cut-off was chosen because it approximates median split of the population; 4 inhalations·day^−1^ corresponds to finishing a 200-inhalation canister within 2 months.

Differences in exacerbation rates between treatments within each SABA use group were compared by calculating rate ratios adjusted for baseline post-bronchodilator % predicted FEV_1_, baseline COPD exacerbation history (1 or ≥2 in the past year), log baseline blood eosinophil count, region and ICS use at screening (yes or no) using negative binomial regression; logarithm of the time at risk of experiencing an exacerbation was an offset variable. Analyses were supplemented with generalised additive models (GAMs) predicting exacerbation rates using mean baseline SABA inhalations·day^−1^ as a continuous variable.

#### Analysis 2: exacerbation rates and SGRQ total score change by post-randomisation rescue SABA use

For assessment of moderate/severe and severe exacerbation rates over 52 weeks and change from baseline SGRQ total score over 24 weeks by post-randomisation rescue SABA use, participants were categorised using the same cut-offs described for analysis 1 (≤4 *versus* >4 inhalations·day^−1^).

Exacerbation rate differences between treatments within each SABA use group were compared as described for analysis 1. Differences in change from baseline SGRQ total score over 24 weeks between treatments were assessed within each post-randomisation rescue SABA group using least squares mean (LSM) from linear repeated measures models including treatment, visit, treatment-by-visit interaction and ICS use at screening (yes or no) as categorical covariates, and log baseline blood eosinophils, baseline SGRQ total score, baseline post-bronchodilator % predicted FEV_1_, and per cent bronchodilator reversibility as continuous covariates. Analyses were supplemented by GAMs predicting exacerbation rates and change from baseline in SGRQ total score using mean post-randomisation rescue SABA inhalations/day as a continuous variable.

As analysis 2 used post-randomisation information and rescue medication use differed by treatment group, there is potential for confounding with treatment.

#### Analysis 3: post-randomisation rescue SABA use surrounding COPD exacerbations

For assessment of post-randomisation rescue SABA use surrounding an exacerbation, rescue SABA use was evaluated 30 days before, the onset day and 30 days after onset of the first moderate/severe, moderate and severe exacerbation.

Analyses focused on the first exacerbation to ensure participants were only considered once. Exacerbation onset date was the earliest of: medication start date (systemic corticosteroid and/or antibiotic); hospitalisation start date (if applicable); or death date (if relevant). Participants were included regardless of whether the exacerbation onset date was within the first 30 days of the study or if they discontinued within 30 days of the exacerbation onset date.

As analysis 3 used post-randomisation information and rescue SABA use differed by treatment, there is potential for confounding with treatment. Therefore, analyses focused on overall results across treatments, rather than on comparing treatments.

ETHOS was not prospectively powered for these *post hoc* analyses. As such, reported p-values are unadjusted for multiplicity and provided for descriptive purposes only. Results are presented for BGF 320 (referred to as BGF hereafter) *versus* GFF or BFF, with BGF 160 comparisons reported in supplementary tables S1–S4 and figures S1–S4).

## Results

### Patient disposition

Participant disposition has previously been described [9]. In brief, 8588 participants were randomised and 8573 received study treatment. Of 8509 participants in the mITT population (those with post-randomisation data obtained before treatment discontinuation), 5639 (66.3%) had mean baseline rescue SABA use ≥1.0 inhalation·day^−1^ and constituted the rescue SABA use population. One participant (BGF 160/14.4/10 µg) was excluded from the *post hoc* analyses because their evening rescue dose information at baseline was unknown.

### Exacerbation rates by baseline rescue SABA use

Table 1 reports baseline demographic and clinical characteristics by baseline SABA use and treatment*.* In total, 3074 participants used ≤4 inhalations·day^−1^ and 2564 used >4 inhalations·day^−1^ of SABA at baseline. Characteristics were generally comparable between SABA use groups and were well balanced across treatments within SABA use groups.

**TABLE 1:** Baseline demographics and clinical characteristics by baseline rescue short-acting β_2_-agonist (SABA) use level and treatment (BGF 320/14.4/10 µg, GFF 14.4/10 µg, BFF 320/10 µg)^#^

	≤4 inhalations·day^−1^ SABA			>4 inhalations·day^−1^ SABA		
	BGF	GFF	BFF	BGF	GFF	BFF
**Participants, n**	797	757	773	633	632	656
**Age years, mean±sd**	64.5±7.7	64.9±7.5	65.2±7.6	64.0±7.6	64.0±7.8	63.7±7.7
**Sex, n (%)**						
Female	333 (41.8)	299 (39.5)	321 (41.5)	285 (45.0)	298 (47.2)	276 (42.1)
Male	464 (58.2)	458 (60.5)	452 (58.5)	348 (55.0)	334 (52.8)	380 (57.9)
**Current smoker, n (%)**	326 (40.9)	275 (36.3)	299 (38.7)	290 (45.8)	295 (46.7)	288 (43.9)
**Moderate/severe exacerbations in previous year, n (%)**						
1	347 (43.5)	350 (46.2)	333 (43.1)	295 (46.6)	284 (44.9)	292 (44.5)
≥2	450 (56.5)	407 (53.8)	440 (56.9)	338 (53.4)	348 (55.1)	364 (55.5)
**Blood eosinophil count, n (%)**						
≥150 cells·mm^−3^	483 (60.6)	461 (60.9)	460 (59.5)	399 (63.0)	405 (64.1)	425 (64.8)
≥300 cells·mm^−3^	109 (13.7)	92 (12.2)	112 (14.5)	116 (18.3)	122 (19.3)	126 (19.2)
**FEV_1_ % predicted, mean±sd^¶^**	43.4±10.1	43.2±10.2	43.5±10.3	41.1±10.3	40.9±9.9	40.1±10.0
**Post-bronchodilator % reversibility, mean±sd**	15.4±16.2	16.2±16.9	14.5±14.8	18.1±17.2	18.0±16.5	17.4±16.7
**SGRQ score, mean±sd**	51.5±15.6	49.9±15.4	50.0±16.1	56.8±15.6	57.0±15.5	56.9±15.7
**CAT score, mean±sd**	19.8±6.3	19.1±6.3	19.3±6.1	21.8±6.3	21.9±6.4	21.6±6.5
**Used ICS at screening, n (%)**	665 (83.4)	614 (81.1)	632 (81.8)	506 (79.9)	514 (81.3)	509 (77.6)
**SABA use inhalations·day^−1^, mean±sd**	2.5±1.0	2.5±1.0	2.5±1.0	7.2±2.9	7.1±2.7	7.2±2.7

BGF: budesonide/glycopyrronium/formoterol fumarate dihydrate; GFF: glycopyrronium/formoterol fumarate dihydrate; BFF: budesonide/formoterol fumarate dihydrate; FEV_1_: forced expiratory volume in 1 s; SGRQ: St George's Respiratory Questionnaire; CAT: COPD Assessment Test; ICS: inhaled corticosteroids. ^#^: BGF 160/14.4/10 µg treatment arm data reported in supplementary table S1. ^¶^: baseline defined as the mean of the 30- and 60-min values prior to dosing on Day 1 (Visit 4), if available; otherwise, the mean of the 30- and 60-min pre-bronchodilator assessments at Visit 3 was used, if available; otherwise, the mean of the 30- and 60-min pre-bronchodilator assessments at Visit 2 was used.

**FIGURE 1:**
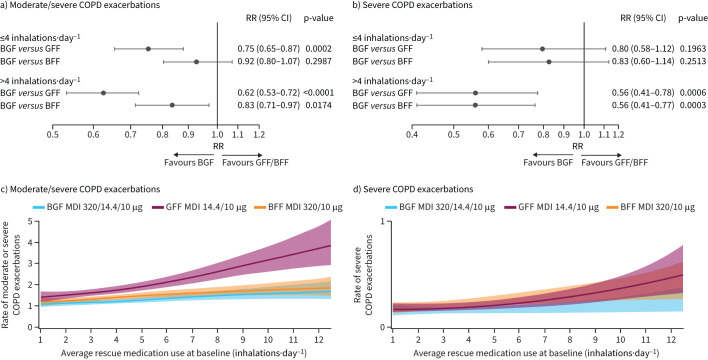
a, c) Moderate/severe and b, d) severe COPD exacerbation rates by baseline rescue short-acting β_2_-agonist (SABA) use and treatment (BGF 320/14.4/10 µg, GFF 14.4/10 µg, BFF 320/10 µg).^#,¶^ Data from generalised additive models. Banded areas denote 95% credible intervals. BFF: budesonide/formoterol fumarate dihydrate; BGF: budesonide/glycopyrronium/formoterol fumarate dihydrate; GFF: glycopyrronium/formoterol fumarate dihydrate; RR: rate ratio; MDI: metered dose inhaler. ^#^: BGF 160/14.4/10 µg treatment arm data reported in supplementary figure S1; ^¶^: treatments were compared adjusting for baseline post-bronchodilator % predicted forced expiratory volume in 1 s (FEV_1_), baseline COPD exacerbation history (1 or ≥2 in the past year), log baseline blood eosinophil count, region and inhaled corticosteroid (ICS) use at screening (yes or no) using negative binomial regression; logarithm of the time at risk of experiencing an exacerbation was an offset variable.

Across treatments, moderate/severe exacerbation rates were numerically higher in participants with higher baseline SABA use (table 2). Benefits of BGF *versus* GFF and BFF on moderate/severe exacerbations were generally seen across baseline SABA use groups, with trends towards greater benefit in participants with higher baseline SABA use (figure 1a and c). This is most apparent in GFF, where there was a steeper increase in moderate/severe exacerbations with increasing mean baseline SABA use *versus* the ICS-containing treatments (BGF and BFF) where increases were more gradual (figure 1c). Severe exacerbation rates were also generally higher in participants with higher baseline SABA use (table 2), with BGF treatment benefits *versus* GFF and BFF observed among participants with baseline SABA use >4 inhalations·day^−1^ and similar trends with baseline SABA use ≤4 inhalations·day^−1^ (figure 1b and d).

**TABLE 2:** Exacerbation rates by baseline rescue short-acting β_2_-agonist (SABA) use level and treatment (BGF 320/14.4/10 µg, GFF 14.4/10 µg, BFF 320/10 µg)^#^

	≤4 inhalations·day^−1^ SABA			>4 inhalations·day^−1^ SABA		
	BGF	GFF	BFF	BGF	GFF	BFF
**SABA use at baseline**						
Participants, n	797	757	773	633	632	656
Moderate/severe COPD exacerbations						
Participants with exacerbations, n (%)	394 (49.4)	403 (53.2)	392 (50.7)	341 (53.9)	378 (59.8)	385 (58.7)
Adjusted exacerbation rate, mean±se	1.13±0.06	1.51±0.08	1.23±0.07	1.34±0.08	2.18±0.12	1.62±0.09
Severe COPD exacerbations						
Participants with exacerbations, n (%)	81 (10.2)	96 (12.7)	89 (11.5)	71 (11.2)	95 (15.0)	111 (16.9)
Adjusted exacerbation rate, mean±se	0.14±0.02	0.17±0.02	0.17±0.02	0.13±0.02	0.24±0.03	0.24±0.02
**SABA use post-randomisation**						
Participants, n	945	817	849	482	572	578
Moderate/severe COPD exacerbations						
Participants with exacerbations, n (%)	447 (47.3)	429 (52.5)	422 (49.7)	288 (59.8)	352 (61.5)	355 (61.4)
Adjusted exacerbation rate, mean±se	1.04±0.05	1.38±0.07	1.14±0.06	1.60±0.10	2.51±0.15	1.83±0.11
Severe COPD exacerbations						
Participants with exacerbations, n (%)	95 (10.1)	107 (13.1)	92 (10.8)	57 (11.8)	84 (14.7)	108 (18.7)
Adjusted exacerbation rate, mean±se	0.12±0.01	0.17±0.02	0.14±0.02	0.17±0.02	0.24±0.03	0.31±0.03

BGF: budesonide/glycopyrronium/formoterol fumarate dihydrate; GFF: glycopyrronium/formoterol fumarate dihydrate; BFF: budesonide/formoterol fumarate dihydrate. ^#^: BGF 160/14.4/10 µg treatment arm data reported in supplementary table S2 (baseline SABA use) and table S4 (post-randomisation SABA use).

### Exacerbation rates and SGRQ total score change by post-randomisation rescue SABA use

Baseline demographic and clinical characteristics were generally comparable between post-randomisation SABA use groups (≤4 inhalations·day^−1^ (n=3483); >4 inhalations·day^−1^ (n=2150)) and well balanced across treatments within post-randomisation SABA use groups (table 3).

**TABLE 3:** Baseline demographics and clinical characteristics by post-randomisation rescue short-acting β_2_-agonist (SABA) use level and treatment (BGF 320/14.4/10 µg, GFF 14.4/10 µg, BFF 320/10 µg)^#^

	≤4 inhalations·day^−1^ SABA			>4 inhalations·day^−1^ SABA		
	BGF	GFF	BFF	BGF	GFF	BFF
**Participants, n**	945	817	849	482	572	578
**Age years, mean±sd**	64.5±7.6	64.8±7.7	65.0±7.5	63.9±7.7	64.0±7.6	63.9±7.9
**Sex, n (%)**						
Female	408 (43.2)	336 (41.1)	354 (41.7)	209 (43.4)	261 (45.6)	243 (42.0)
Male	537 (56.8)	481 (58.9)	495 (58.3)	273 (56.6)	311 (54.4)	335 (58.0)
**Current smoker, n (%)**	371 (39.3)	317 (38.8)	312 (36.7)	243 (50.4)	253 (44.2)	275 (47.6)
**Moderate/severe exacerbations in previous year, n (%)**						
1	411 (43.5)	378 (46.3)	345 (40.6)	230 (47.7)	256 (44.8)	279 (48.3)
≥2	534 (56.5)	439 (53.7)	504 (59.4)	252 (52.3)	316 (55.2)	299 (51.7)
**Blood eosinophil count, n (%)**						
≥150 cells·mm^−3^	588 (62.2)	489 (59.9)	532 (62.7)	293 (60.8)	377 (65.9)	352 (60.9)
≥300 cells·mm^−3^	145 (15.3)	111 (13.6)	136 (16.0)	79 (16.4)	103 (18.0)	102 (17.6)
**FEV_1_% predicted, mean±sd^¶^**	43.7±10.3	43.5±10.1	43.3±10.2	39.9±9.8	40.3±9.8	39.9±10.1
**Post-bronchodilator % reversibility, mean±sd**	16.7±16.5	16.6±16.8	15.2±15.7	16.3±17.0	17.7±16.7	16.8±15.9
**SGRQ score, mean±sd**	51.2±15.8	50.7±15.8	51.1±16.4	59.0±14.4	56.5±15.3	56.2±15.6
**CAT score, mean±sd**	19.8±6.1	19.5±6.4	19.7±6.2	22.4±6.3	21.6±6.6	21.3±6.5
**Used ICS at screening, n (%)**	768 (81.3)	653 (79.9)	669 (78.8)	400 (83.0)	475 (83.0)	470 (81.3)
**SABA use inhalations·day^−1^, mean±sd**	3.4±2.0	3.3±2.0	3.4±2.2	6.9±3.6	6.4±3.2	6.6±3.2

BGF: budesonide/glycopyrronium/formoterol fumarate dihydrate; GFF: glycopyrronium/formoterol fumarate dihydrate; BFF: budesonide/formoterol fumarate dihydrate; FEV_1_: forced expiratory volume in 1 s; SGRQ: St George's Respiratory Questionnaire; CAT: COPD Assessment Test; ICS: inhaled corticosteroids. ^#^: BGF 160/14.4/10 µg treatment arm data reported in supplementary table S3. ^¶^: baseline defined as the mean of the 30- and 60-min values prior to dosing on Day 1 (Visit 4), if available; otherwise, the mean of the 30- and 60-min pre-bronchodilator assessments at Visit 3 was used, if available; otherwise, the mean of the 30- and 60-min pre-bronchodilator assessments at Visit 2 was used.

**FIGURE 2:**
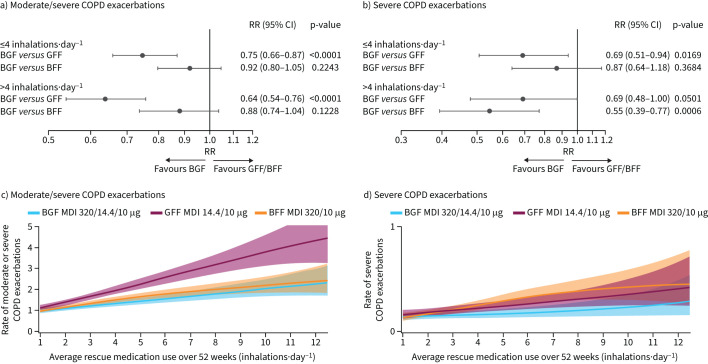
a, c) Moderate/severe and b, d) severe COPD exacerbation rates by post-randomisation rescue short-acting β_2_-agonist (SABA) use and treatment (BGF 320/14.4/10 µg, GFF 14.4/10 µg, BFF 320/10 µg).^#,¶^ Data from generalised additive models. Banded areas denote 95% credible intervals. BFF: budesonide/formoterol fumarate dihydrate; BGF: budesonide/glycopyrronium/formoterol fumarate dihydrate; GFF: glycopyrronium/formoterol fumarate dihydrate; RR: rate ratio; MDI: metered dose inhaler. ^#^: BGF 160/14.4/10 µg treatment arm data reported in supplementary figure S2; ^¶^: treatments were compared adjusting for baseline post-bronchodilator % predicted forced expiratory volume in 1 s (FEV_1_), baseline COPD exacerbation history (1 or ≥2 in the past year), log baseline blood eosinophil count, region and inhaled corticosteroids (ICS) use at screening (yes or no) using negative binomial regression; logarithm of the time at risk of experiencing an exacerbation was an offset variable.

Moderate/severe and severe exacerbation rates were numerically greater in participants with higher post-randomisation SABA use across treatments (table 2). Evidence of BGF benefit *versus* GFF and BFF on moderate/severe exacerbations (figure 2a) and on severe exacerbations (figure 2b) were observed in both post-randomisation SABA groups, with generally numerically larger estimates with >4 inhalations·day^−1^. Viewing on the continuum in the GAMs, increasing treatment differences favouring BGF with greater post-randomisation SABA use were observed on moderate/severe (figure 2c) and severe (figure 2d) exacerbation rates until use exceeded approximately 7 inhalations·day^−1^, where estimates of benefit on moderate/severe exacerbations *versus* BFF started to decrease.

**FIGURE 3:**
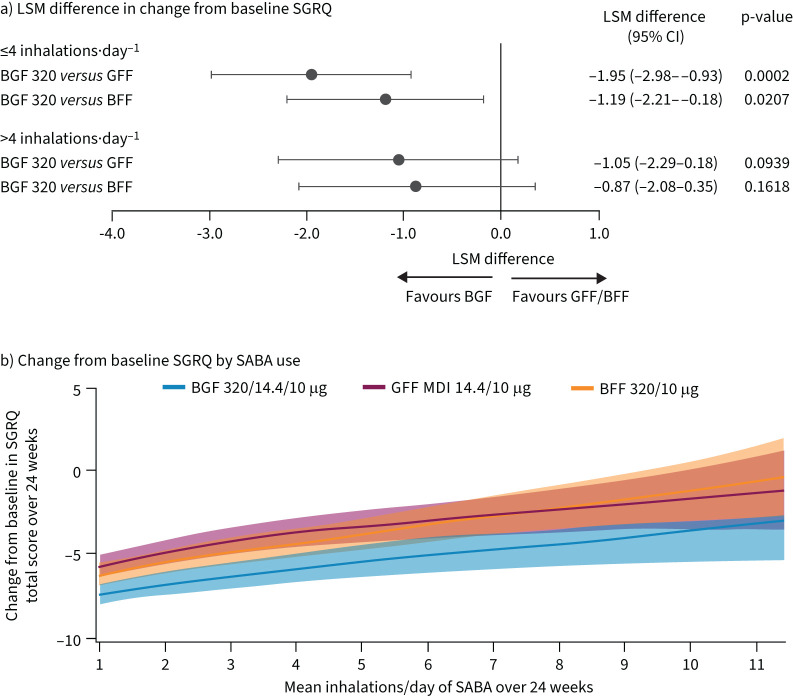
a) Least squares mean (LSM) difference in change from baseline in St George's Respiratory Questionnaire (SGRQ) total score and b) change from baseline in SGRQ total score over 24 weeks by post-randomisation rescue short-acting β_2_-agonist (SABA) use level and treatment (BGF 320/14.4/10 µg, GFF 14.4/10 µg, BFF 320/10 µg).^#,¶,+^ Data from generalised additive models. Banded areas denote 95% credible intervals. BFF: budesonide/formoterol fumarate dihydrate; BGF: budesonide/glycopyrronium/formoterol fumarate dihydrate; GFF: glycopyrronium/formoterol fumarate dihydrate; LSM: least squares mean; MDI: metered dose inhaler. ^#^: BGF 160/14.4/10 µg treatment arm data reported in supplementary figure S3. ^¶^: participants with post-randomisation rescue SABA use ≤4 inhalations·day^−1^: LSM±se was −8.2±0.38 for BGF 320 (n=923), −6.2±0.41 for GFF (n=791) and −7.0±0.40 for BFF (n=829); participants with post-randomisation rescue SABA use >4 inhalations·day^−1^: LSM±se was −4.3±0.49 for BGF 360 (n=467), −3.2±0.47 for GFF (n=527) and −3.4±0.45 for BFF (n=544). ^+^: LSM derived from linear repeated measures models including treatment, visit, treatment-by-visit interaction and inhaled corticosteroid use at screening (yes or no) as categorical covariates, and log baseline blood eosinophils, baseline SGRQ total score, baseline post-bronchodilator % predicted forced expiratory volume in 1 s and % bronchodilator reversibility as continuous covariates.

For SGRQ, estimated BGF benefits *versus* dual therapies were similar in participants with high *versus* low post-randomisation SABA use (figure 3a and b).

### Post-randomisation rescue SABA use surrounding exacerbations

**FIGURE 4:**
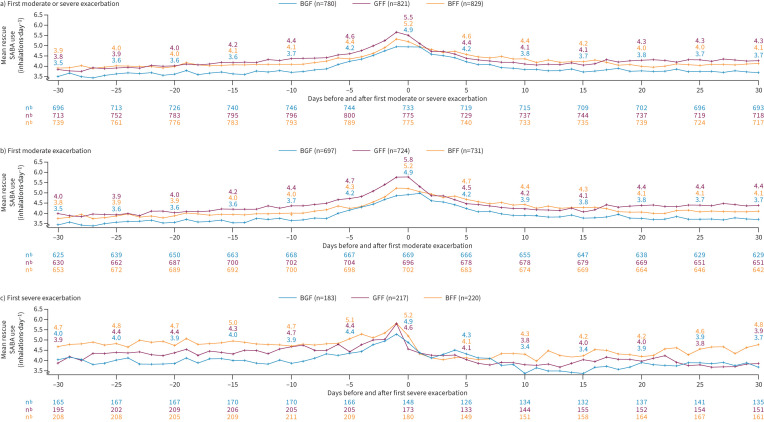
Rescue short-acting β_2_-agonist (SABA) use surrounding a) the first moderate/severe, b) first moderate and c) first severe COPD exacerbation by treatment (BGF 320/14.4/10 µg, GFF 14.4/10 µg, BFF 320/10 µg).^#^ BFF: budesonide/formoterol fumarate dihydrate; BGF: budesonide/glycopyrronium/formoterol fumarate dihydrate; GFF: glycopyrronium/formoterol fumarate dihydrate. ^#^: BGF 160/14.4/10 µg treatment arm data reported in supplementary figure S4. Missing data were for participants who had a first exacerbation but were missing SABA diary counts for that day.

Post-randomisation SABA use increased 30 days before, and decreased 30 days following, the first moderate/severe (figure 4a), first moderate (figure 4b) and first severe exacerbation (figure 4c) across all treatments, with the maximum increase being fewer than 2 inhalations·day^−1^. Increases in SABA use were gradual from Day −30 to Day −7 before exacerbation onset, with increases becoming more pronounced in the 7 days before exacerbation onset of the first moderate/severe and first moderate exacerbations (figure 4a). Decreases in post-randomisation SABA use immediately following exacerbation onset appeared more abrupt for severe than moderate exacerbations (figure 4b and c) in the setting of other treatment factors that were not controlled.

Increases in post-randomisation rescue SABA use were numerically lower with BGF before and after the first moderate/severe (figure 4a) and moderate exacerbation (figure 4b). Similar trends were seen for severe exacerbations, although there were relatively few events making observations less clear (figure 4c).

## Discussion

These *post hoc* analyses of ETHOS examined relationships between treatment and rescue SABA use on exacerbation rates and HRQoL. The findings indicate higher rescue SABA use (>4 *versus* ≤4 inhalations·day^−1^) tended to be associated with elevated exacerbation rates, regardless of whether use was categorised at baseline or post-randomisation. This is reflected in the GAM plots for moderate/severe exacerbations, where rates in each arm increase as average SABA use increases (for baseline and on-treatment use), with generally greater separation for BGF *versus* dual therapies with higher rescue medication use. Additionally, these findings suggest baseline SABA use was a better exacerbation predictor in GFF (and possible guide to treatment escalation). Further, increased rescue SABA use also was an indicator of an exacerbation, with use increasing as early as 30 days before exacerbation onset. Lastly, evidence of BGF benefit *versus* dual therapy on exacerbation rates was observed across SABA use level. Taken together, these findings build on the existing literature by providing data on peri-exacerbation risk and its timeframe and demonstrating BGF reduces exacerbation risk over dual therapy regardless of SABA use level.

These findings demonstrating that higher rescue SABA use was generally associated with increased COPD exacerbation risk align with previous reports in people with COPD [3, 6, 8, 13, 14] and people with asthma [15]. In a retrospective study, the first occurrence of SABA use predicted exacerbations within 3 weeks in people with COPD [3]. In the same study, exacerbation risk increased with increasing SABA use, with 10-month exacerbation risk progressively increasing in people with mean SABA use of 2–5, 6–9 and >10 inhalations·day^−1^ *versus* <2 inhalations·day^−1^ [3]. In a population-based cross-sectional survey of people with COPD, more frequent reliever use (≥1 occasion/week *versus* <1 occasion/week) was also associated with higher moderate or severe exacerbation rates [6]. A previous predictive modelling analysis based on seven studies, including ETHOS, examined predictors of moderate/severe exacerbations [13]. In the final model, increased baseline rescue medication inhalations/day was a significant predictor of exacerbation risk [13]. The current *post hoc* analyses expand on these findings by demonstrating not only that baseline rescue medication use is an important prognostic covariate of exacerbation risk, but that post-randomisation rescue medication use is as well. Although, it should also be considered that SABA use is a consequence of symptomatic instability.

In considering the analyses by SABA use level, it is important to understand the stability of the SABA use phenotype. To assess SABA use phenotype stability, data during the 4-week screening period was analysed using a transition plot. During Week ‒4 and Week ‒3 of screening, there were a large number of unknown values for mean daily number of SABA inhalations because not all participants required longer screening periods. However, analysis of Week −2 and Week −1, where data were available for the majority of participants with known SABA use frequency, showed that the SABA use phenotype remained stable during this period (see supplementary figure S5).

The current analyses provide insight into rescue medication use changes in the days surrounding an exacerbation. Overall, these findings show that increasing rescue medication use may identify an impending exacerbation. While prospective studies are needed, the patterns of increased rescue SABA use preceding an exacerbation may facilitate earlier detection and intervention, which could reduce exacerbation severity and permit earlier treatment initiation, which has been associated with more rapid recovery [16]. It could also prompt review of patients’ overall management to prevent future exacerbations. It should be noted that decreases in post-randomisation rescue SABA use immediately following exacerbation onset appeared more abrupt with severe exacerbations, possibly because other interventions (including nebulised SABA) were provided to hospitalised participants following severe exacerbations or due to SABA use not being recorded during hospitalisation.

Although treatment differences should be interpreted cautiously, evidence of BGF benefit *versus* dual therapies on exacerbation rates were observed across SABA use groups. Benefits of BGF *versus* dual therapies on moderate/severe and severe exacerbation rates were generally seen in both low and high baseline rescue SABA use, with trends towards greater benefits observed with higher baseline SABA use. Similarly, evidence of BGF benefits *versus* dual therapies on exacerbation rates were seen in both post-randomisation rescue SABA use groups, with trends towards greater benefits observed in participants with higher post-randomisation SABA use. Although mean rescue SABA use increased across treatment arms as an exacerbation approached, increases were lower in magnitude with BGF before and after the first moderate/severe and the first moderate exacerbation. These findings suggest that those with high rescue SABA use may derive greater benefit from BGF triple therapy *versus* dual therapies. However, it should be noted that individuals on triple therapy already experience fewer exacerbations, and hence use less SABA overall.

The current analysis also evaluated HRQoL using SGRQ total score change from baseline. Improvement in HRQoL was not associated with SABA use level across treatments, with post-randomisation SGRQ total score improvements being similar regardless of post-randomisation rescue SABA use level. In an analysis from the Early MAXimisation of bronchodilation for improving COPD stability (EMAX) trial, people with COPD with greater baseline SABA use had worse HRQoL, as measured by higher baseline SGRQ total score, though it should be noted that the cut-off for SABA use was ≥1.5 inhalations·day^−1^ in the EMAX trial *versus* >4 inhalations·day^−1^ in our analyses [5]. In a systematic review of rescue medication use as a COPD symptom marker, increases in baseline SABA use were associated with increases in SGRQ total score, indicating greater rescue medication use was associated with worse HRQoL [7]. Whilst treatment differences should be interpreted with caution, in the current analysis BGF improved SGRQ *versus* dual therapies similarly with both lower and higher post-randomisation SABA use.

There are limitations in the current analyses. First, ETHOS was not prospectively powered for these *post hoc* analyses. As such, reported p-values are provided for descriptive purposes only. Second, some analyses used post-randomisation information and rescue medication use differed by treatment group so there is potential confounding with treatment. Third, the number of severe exacerbations was relatively small, resulting in higher variability and making data interpretation challenging. Fourth, as SABA use was self-reported using electronic diaries, the possibility that reporting bias influenced SABA use level categorisations should be acknowledged. Finally, participants with severe exacerbations were hospitalised and may have received other interventions, including nebulised SABA, which were not captured.

In conclusion, these *post hoc* analyses indicate higher rescue SABA use is an important prognostic covariate of increased COPD exacerbation risk, and increased rescue SABA use can be an indicator of an impending exacerbation and poorly controlled COPD. Based on these findings, increases in SABA use could be indicative of an impending exacerbation within 7 days. With this in mind, exacerbation monitoring can be incorporated into future digital patient care algorithms (*i.e*., e-diaries) and prospectively studied in intelligent study designs. Further, BGF benefits on exacerbation rates and HRQoL *versus* dual therapies were observed across high and low SABA use levels, with treatment effects on exacerbations being generally more pronounced with higher baseline rescue SABA use. This suggests people with high baseline rescue medication use (>4 inhalations·day^−1^ in this study) may derive greater benefit from additional therapeutic interventions, including escalation to triple therapy.

## Supplementary material

**Please note:** supplementary material is not edited by the Editorial Office, and is uploaded as it has been supplied by the author. Supplementary material 00348-2025.SUPPLEMENT
