# Supplementary material for: Short-acting β2-agonist use, exacerbation risk and triple therapy in COPD: post hoc analyses of ETHOS
Source: ERJ Open Res. 2025 Dec 15;11(6):00348-2025. doi: 10.1183/23120541.00348-2025 (PMC12704147; doi:10.1183/23120541.00348-2025)
Supplement: Supplementary file 1 [file 00348-2025.SUPPLEMENT.pdf]

SUPPLEMENTARY INFORMATION

Table S1. Baseline demographics and clinical characteristics by baseline rescue SABA use level and treatment (BGF 160/14.4/10 µg, GFF 14.4/10 µg, BFF 160/10 µg)[a]

	≤4 inhalations/day SABA			>4 inhalations/day SABA		
	**BGF**	**GFF**	**BFF**	**BGF**	**GFF**	**BFF**
N	747	757	773	643	632	656
Age, years, mean (SD)	65.1 (7.4)	64.9 (7.5)	65.2 (7.6)	63.7 (7.7)	64.0 (7.8)	63.7 (7.7)
Sex, n (%)						
Female	305 (40.8)	299 (39.5)	321 (41.5)	257 (40.0)	298 (47.2)	276 (42.1)
Male	442 (59.2)	458 (60.5)	452 (58.5)	386 (60.0)	334 (52.8)	380 (57.9)
Current smoker, n (%)	294 (39.4)	275 (36.3)	299 (38.7)	268 (41.7)	295 (46.7)	288 (43.9)
Moderate/severe exacerbations in previous year, n (%)						
1	331 (44.3)	350 (46.2)	333 (43.1)	300 (46.7)	284 (44.9)	292 (44.5)
≥2	416 (55.7)	407 (53.8)	440 (56.9)	343 (53.3)	348 (55.1)	364 (55.5)
Blood eosinophil count, n (%)						
≥150 cells/mm^3	449 (60.1)	461 (60.9)	460 (59.5)	404 (62.8)	405 (64.1)	425 (64.8)
≥300 cells/mm^3	119 (15.9)	92 (12.2)	112 (14.5)	107 (16.6)	122 (19.3)	126 (19.2)
FEV_1 % predicted, mean (SD)[b]	43.2 (10.1)	43.2 (10.2)	43.5 (10.3)	40.4 (10.2)	40.9 (9.9)	40.1 (10.0)
Post-bronchodilator % reversibility, mean (SD)	15.7 (15.4)	16.2 (16.9)	14.5 (14.8)	16.7 (16.6)	18.0 (16.5)	17.4 (16.7)
SGRQ score, mean (SD)	51.5 (16.5)	49.9 (15.4)	50.0 (16.1)	56.9 (15.7)	57.0 (15.5)	56.9 (15.7)
CAT score, mean (SD)	19.9 (6.3)	19.1 (6.3)	19.3 (6.1)	21.3 (6.3)	21.9 (6.4)	21.6 (6.5)
Used ICS at screening, n (%)	623 (83.4)	614 (81.1)	632 (81.8)	513 (79.8)	514 (81.3)	509 (77.6)
SABA use, mean inhalations/day (SD)	2.5 (0.9)	2.5 (1.0)	2.5 (1.0)	7.0 (2.5)	7.1 (2.7)	7.2 (2.7)

[a]1 participant in the BGF treatment arm is excluded from this table and all subsequent analyses as their evening rescue dose information at baseline is unknown.
[b]Baseline defined as the mean of the 30- and 60-minute values prior to dosing on Day 1 (Visit 4), if available; otherwise, the mean of the 30- and 60-minute pre-bronchodilator assessments at Visit 3 was used, if available; otherwise, the mean of the 30- and 60-minute pre-bronchodilator assessments at Visit 2 was used.
BFF, budesonide/formoterol fumarate dihydrate; BGF, budesonide/glycopyrronium/formoterol fumarate dihydrate; CAT, COPD Assessment Test; COPD, chronic obstructive pulmonary disease; FEV_1, forced expiratory volume in 1 second; GFF, glycopyrronium/formoterol fumarate dihydrate; ICS, inhaled corticosteroids; SABA, short-acting $ß_2$-agonist; SD, standard deviation; SGRQ, St George's Respiratory Questionnaire.

Table S2. Exacerbation rates by baseline rescue SABA use level and treatment (BGF 160/14.4/10 µg, GFF 14.4/10 µg, BFF 160/10 µg)

	**≤4 inhalations/day SABA**			**>4 inhalations/day SABA**		
	**BGF**	**GFF**	**BFF**	**BGF**	**GFF**	**BFF**
N	747	757	773	643	632	656
Moderate/severe COPD exacerbations						
Participants with exacerbations, n (%)	356 (47.7)	403 (53.2)	392 (50.7)	367 (57.1)	378 (59.8)	385 (58.7)
Adjusted exacerbation rate (SE)	1.06 (0.06)	1.51 (0.08)	1.23 (0.07)	1.52 (0.08)	2.18 (0.12)	1.62 (0.09)
Severe COPD exacerbations						
Participants with exacerbations, n (%)	76 (10.2)	96 (12.7)	89 (11.5)	97 (15.1)	95 (15.0)	111 (16.9)
Adjusted exacerbation rate (SE)	0.12 (0.02)	0.17 (0.02)	0.17 (0.02)	0.19 (0.02)	0.24 (0.03)	0.24 (0.02)

BFF, budesonide/formoterol fumarate dihydrate; BGF, budesonide/glycopyrronium/formoterol fumarate dihydrate; COPD, chronic obstructive pulmonary disease; GFF, glycopyrronium/formoterol fumarate dihydrate; SABA, short-acting $ß_2$-agonist; SE, standard error.

Table S3. Baseline demographics and clinical characteristics by post-randomisation rescue SABA use level and treatment (BGF 160/14.4/10 µg, GFF 14.4/10 µg, BFF 160/10 µg)

	≤4 inhalations/day SABA			>4 inhalations/day SABA		
	**BGF**	**GFF**	**BFF**	**BGF**	**GFF**	**BFF**
N	872	817	849	518	572	578
Age, years, mean (SD)	64.8 (7.6)	64.8 (7.7)	65.0 (7.5)	63.8 (7.5)	64.0 (7.6)	63.9 (7.9)
Sex, n (%)						
Female	362 (41.5)	336 (41.1)	354 (41.7)	200 (38.6)	261 (45.6)	243 (42.0)
Male	510 (58.5)	481 (58.9)	495 (58.3)	318 (61.4)	311 (54.4)	335 (58.0)
Current smoker, n (%)	350 (40.1)	317 (38.8)	312 (36.7)	212 (40.9)	253 (44.2)	275 (47.6)
Moderate/severe exacerbations in previous year, n (%)						
1	385 (44.2)	378 (46.3)	345 (40.6)	245 (47.3)	256 (44.8)	279 (48.3)
≥2	487 (55.8)	439 (53.7)	504 (59.4)	273 (52.7)	316 (55.2)	299 (51.7)
Blood eosinophil count, n (%)						
≥150 cells/mm^3	530 (60.8)	489 (59.9)	532 (62.7)	323 (62.4)	377 (65.9)	352 (60.9)
≥300 cells/mm^3	138 (15.8)	111 (13.6)	136 (16.0)	89 (17.2)	103 (18.0)	102 (17.6)
FEV_1 % predicted, mean (SD)[a]	43.2 (10.2)	43.5 (10.1)	43.3 (10.2)	39.7 (10.0)	40.3 (9.8)	39.9 (10.1)
Post-bronchodilator % reversibility, mean (SD)	15.9 (16.0)	16.6 (16.8)	15.2 (15.7)	16.5 (16.0)	17.7 (16.7)	16.8 (15.9)
SGRQ score, mean (SD)	52.4 (16.5)	50.7 (15.8)	51.1 (16.4)	56.6 (15.8)	56.5 (15.3)	56.2 (15.6)
CAT score, mean (SD)	20.1 (6.3)	19.5 (6.4)	19.7 (6.2)	21.3 (6.4)	21.6 (6.6)	21.3 (6.5)
Used ICS at screening, n (%)	710 (81.4)	653 (79.9)	669 (78.8)	426 (82.2)	475 (83.0)	470 (81.3)
SABA use, mean inhalations/day (SD)	3.4 (2.1)	3.3 (2.0)	3.4 (2.2)	6.6 (3.1)	6.4 (3.2)	6.6 (3.2)

[a]Baseline defined as the mean of the 30- and 60-minute values prior to dosing on Day 1 (Visit 4), if available; otherwise, the mean of the 30- and 60-minute pre-bronchodilator assessments at Visit 3 was used, if available; otherwise, the mean of the 30- and 60-minute pre-bronchodilator assessments at Visit 2 was used.
BFF, budesonide/formoterol fumarate dihydrate; BGF, budesonide/glycopyrronium/formoterol fumarate dihydrate; CAT, COPD Assessment Test; COPD, chronic obstructive pulmonary disease; FEV_1, forced expiratory volume in 1 second; GFF, glycopyrronium/formoterol fumarate dihydrate; ICS, inhaled corticosteroids; SABA, short-acting ß$_2$-agonist; SD, standard deviation; SGRQ, St George's Respiratory Questionnaire.

Table S4. Exacerbation rates by post-randomisation rescue SABA level and treatment (BGF 160/14.4/10 µg, GFF 14.4/10 µg, BFF 16/10 µg)

	**≤4 inhalations/day SABA**			**>4 inhalations/day SABA**		
	**BGF**	**GFF**	**BFF**	**BGF**	**GFF**	**BFF**
N	872	817	849	518	572	578
Moderate/severe COPD exacerbations						
Participants with exacerbations, n (%)	411 (47.1)	429 (52.5)	422 (49.7)	313 (60.4)	352 (61.5)	355 (61.4)
Adjusted exacerbation rate (SE)	0.98 (0.05)	1.38 (0.07)	1.14 (0.06)	1.78 (0.11)	2.51 (0.15)	1.83 (0.11)
Severe COPD exacerbations						
Participants with exacerbations, n (%)	98 (11.2)	107 (13.1)	92 (10.8)	76 (14.7)	84 (14.7)	108 (18.7)
Adjusted exacerbation rate (SE)	0.12 (0.01)	0.17 (0.02)	0.14 (0.02)	0.20 (0.03)	0.24 (0.03)	0.31 (0.03)

BFF, budesonide/formoterol fumarate dihydrate; BGF, budesonide/glycopyrronium/formoterol fumarate dihydrate; COPD, chronic obstructive pulmonary disease; GFF, glycopyrronium/formoterol fumarate dihydrate; SABA, short-acting $ß_2$-agonist; SE, standard error.

Figure S1. Moderate/severe (a and c) and severe (b and d) COPD exacerbation rates by baseline rescue SABA use and treatment (BGF

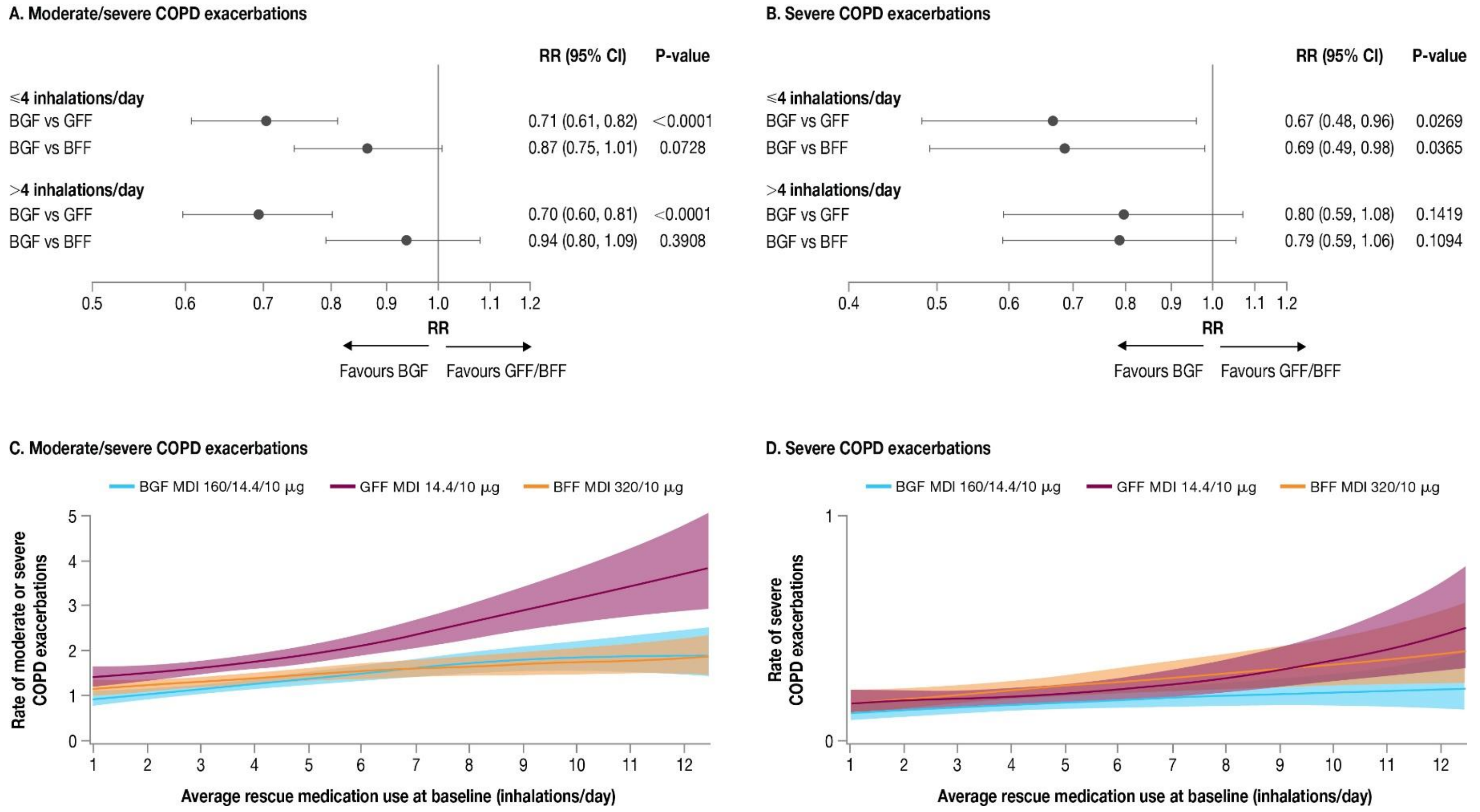

160/14.4/10 µg, GFF 14.4/10 µg, BFF 160/10 µg)[a]

[a]Treatments were compared adjusting for baseline post-bronchodilator percent predicted FEV_1, baseline COPD exacerbation history (1 or ≥2 in the past year), log baseline blood eosinophil count, region, and ICS use at screening (yes or no) using negative binomial regression; logarithm of the time at risk of experiencing an exacerbation was an offset variable.

Data from generalised additive models. Banded areas denote 95% credible intervals.

BFF, budesonide/formoterol fumarate dihydrate; BGF, budesonide/glycopyrronium/formoterol fumarate dihydrate; CI, confidence interval; COPD, chronic obstructive pulmonary disease; FEV_1; forced expiratory volume in 1 second; GFF, glycopyrronium/formoterol fumarate dihydrate; ICS, inhaled corticosteroids; RR, rate ratio; SABA, short-acting $ß_2$-agonist.

Figure S2. Moderate/severe (a and c) and severe (b and d) exacerbation rates by post-randomisation rescue SABA use and treatment (BGF

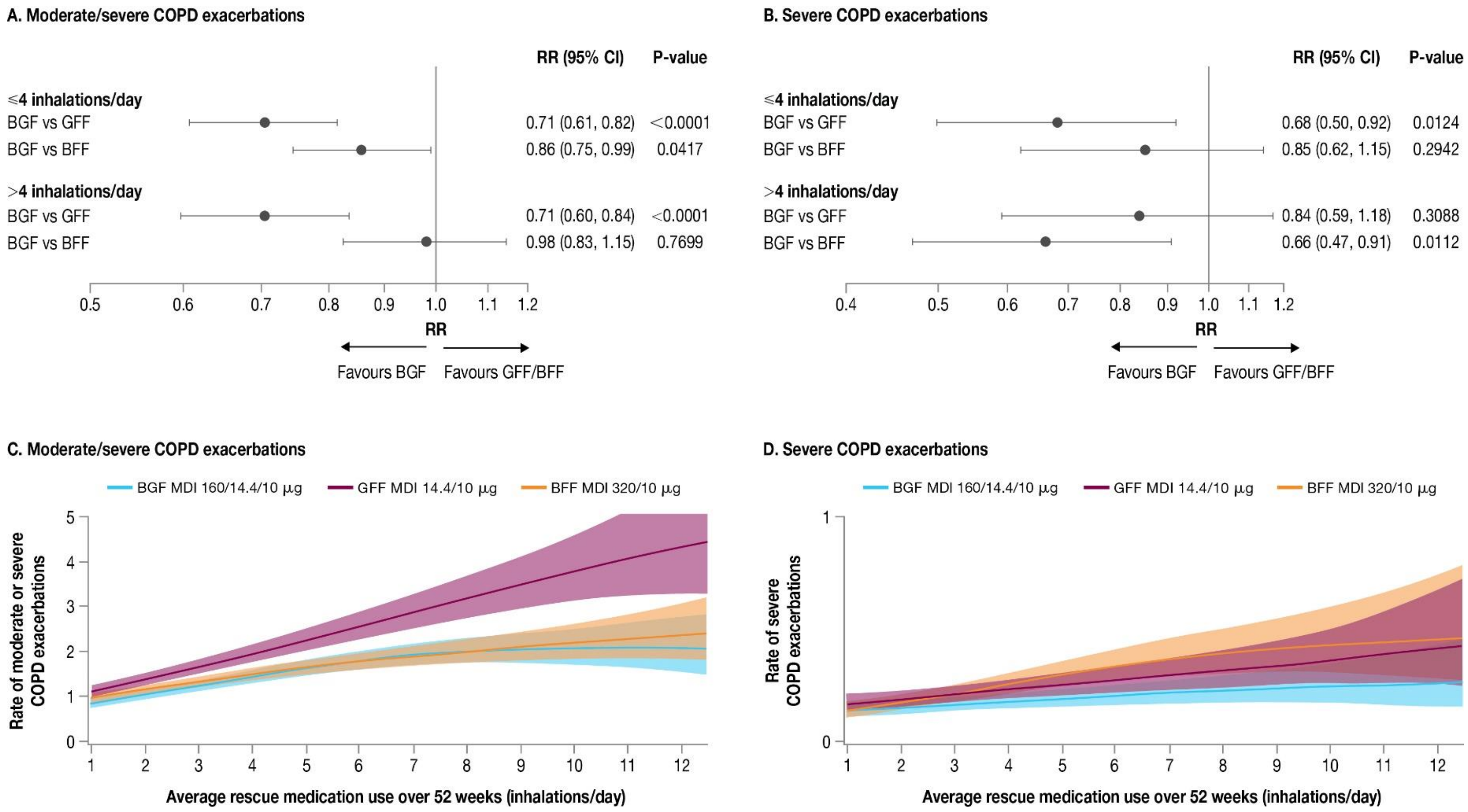

160/14.4/10 µg, GFF 14.4/10 µg, BFF 160/10 µg)[a]

[a]Treatments were compared adjusting for baseline post-bronchodilator percent predicted FEV_1, baseline COPD exacerbation history (1 or ≥2 in the past year), log baseline blood eosinophil count, region, and ICS use at screening (yes or no) using negative binomial regression; logarithm of the time at risk of experiencing an exacerbation was an offset variable.

Data from generalised additive models. Banded areas denote 95% credible intervals.

BFF, budesonide/formoterol fumarate dihydrate; BGF, budesonide/glycopyrronium/formoterol fumarate dihydrate; CI, confidence interval; COPD, chronic obstructive pulmonary disease; FEV_1, forced expiratory volume in 1 second; GFF, glycopyrronium/formoterol fumarate dihydrate; ICS, inhaled corticosteroids; RR, rate ratio; SABA, short-acting ß$_2$-agonist.

Figure S3. Change from baseline in SGRQ total score over 24 weeks by post-randomisation rescue SABA use level and treatment (BGF 160/14.4/10 µg, GFF 14.4/10 µg, BFF 160/10 µg)[a,b]

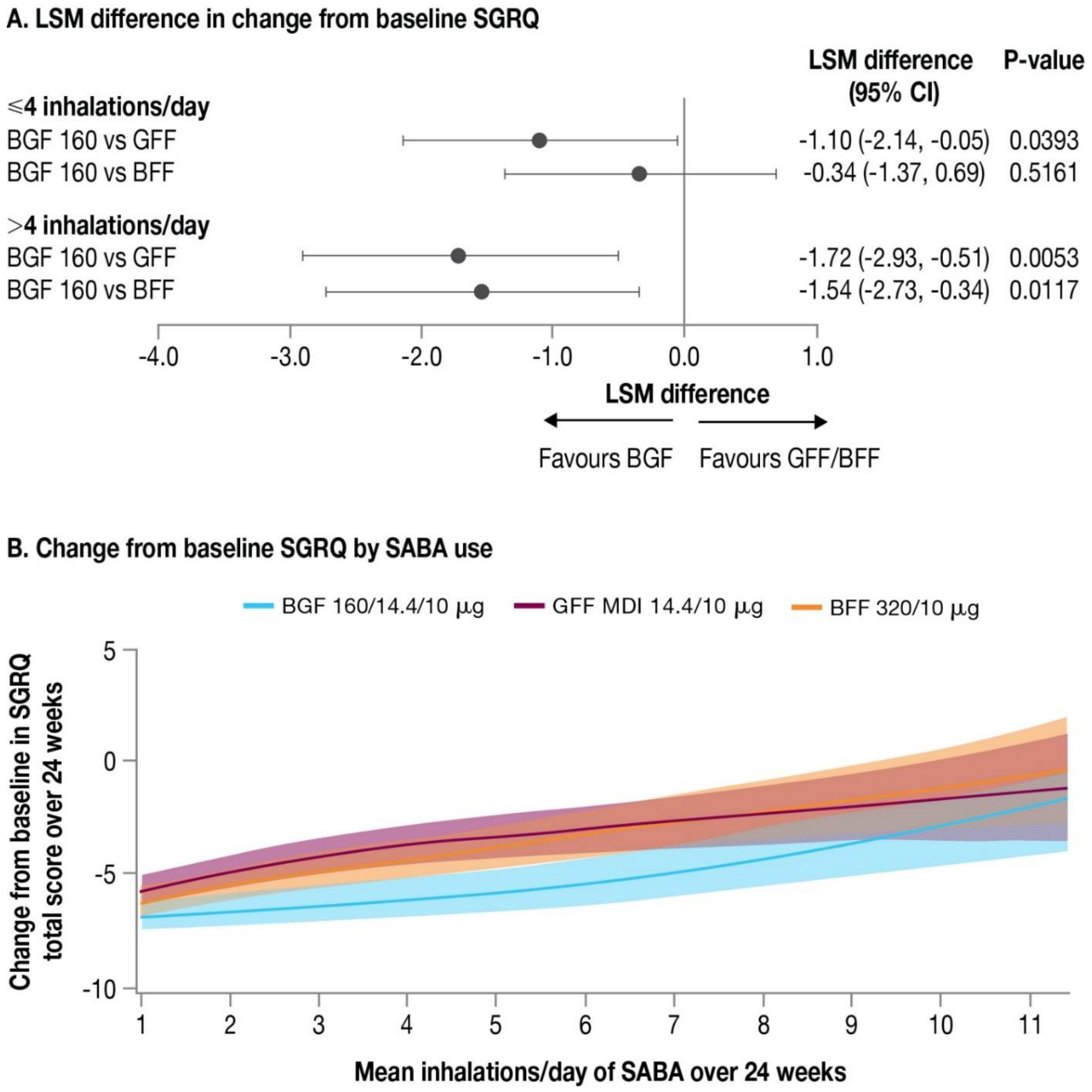

[a]Participants with post-randomisation rescue SABA use ≤4 inhalations/day: LSM (SE) was–7.3 (0.40) for BGF (N=849), –6.2 (0.41) for GFF (N=791), and –7.0 (0.40) for BFF (N=829); participants with post-randomisation rescue SABA use >4 inhalations/day: –5.0 (0.47) for BGF (N=498), –3.2 (0.47) for GFF (N=527), and –3.4 (0.45) for BFF (N=544).

[b]LSM derived from linear repeated measures models including treatment, visit, treatment-by-visit interaction, and ICS use at screening (yes or no) as categorical covariates, and log baseline blood eosinophils, baseline SGRQ total score, baseline post-bronchodilator percent predicted FEV_1, and percent bronchodilator reversibility as continuous covariates.

Data from generalised additive models. Banded areas denote 95% credible intervals.

BFF, budesonide/formoterol fumarate dihydrate; BGF, budesonide/glycopyrronium/formoterol fumarate dihydrate; CI, confidence interval; FEV_1, forced expiratory volume in 1 second; GFF, glycopyrronium/formoterol fumarate dihydrate µg; ICS, inhaled corticosteroids; LSM, least squares mean; short-acting $ß_2$-agonist; SE, standard error; SGRQ, St George's Respiratory Questionnaire.

Figure S4. Rescue SABA use surrounding the first moderate/severe (a), first moderate (b), and first severe (c) COPD exacerbation by treatment (BGF 160/14.4/10 µg, GFF 14.4/10 µg, BFF 160/10 µg)

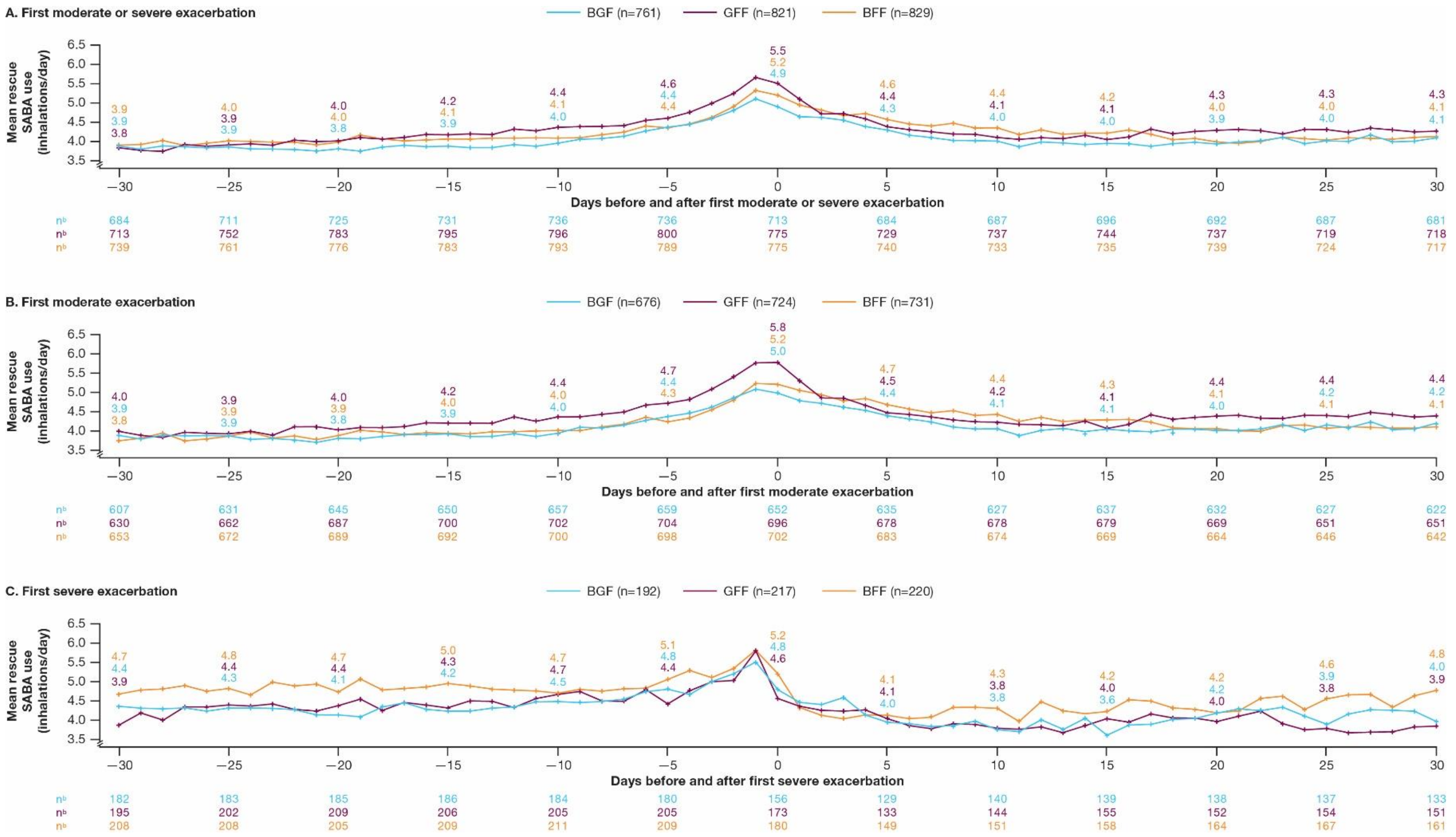

Missing data were for participants who had a first exacerbation but were missing SABA diary counts for that day.
BFF, budesonide/formoterol fumarate dihydrate; BGF, budesonide/glycopyrronium/formoterol fumarate dihydrate; GFF, glycopyrronium/formoterol fumarate dihydrate; mITT, modified intent-to-treat; SABA, short-acting ß$_2$-agonist.

Figure S5. Rescue SABA use phenotype stability

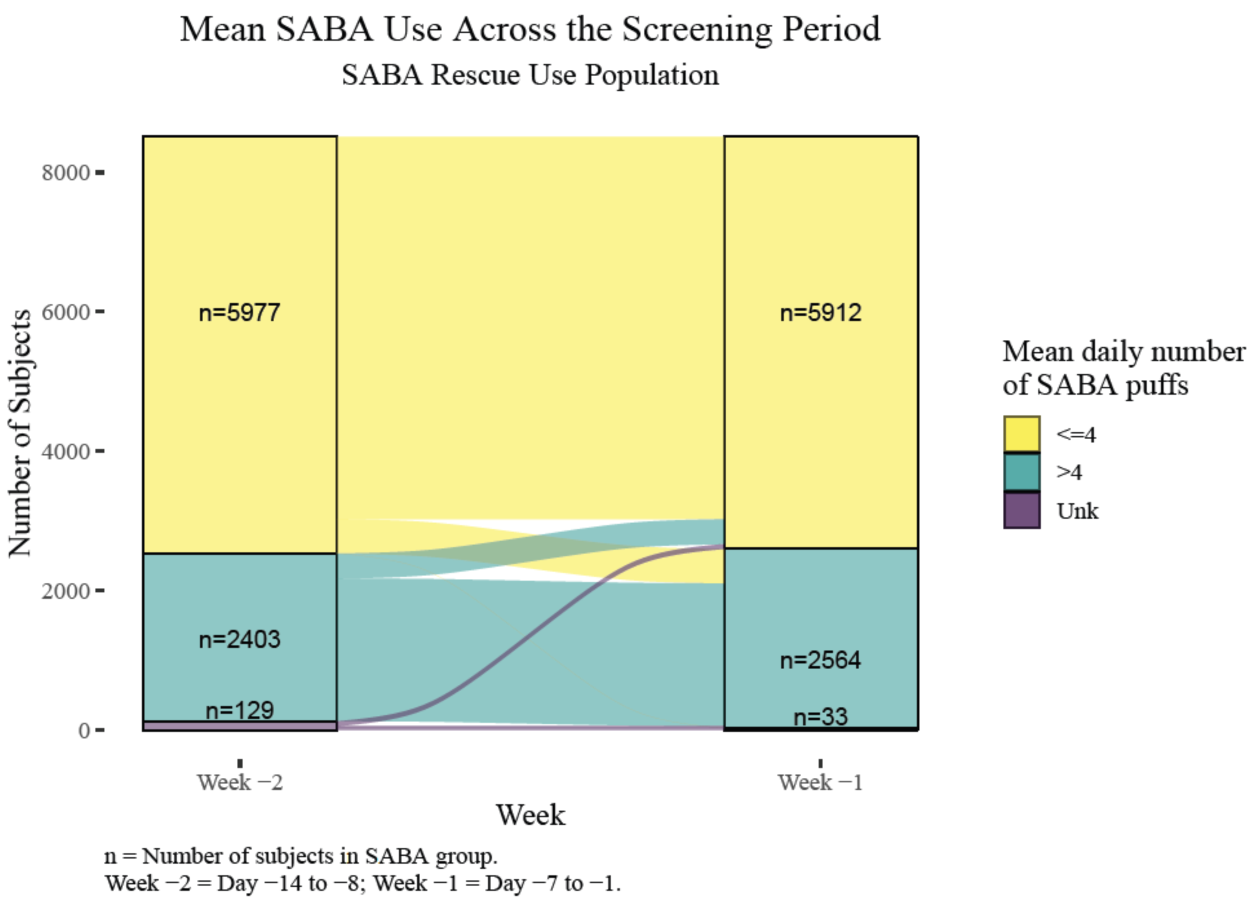

SABA, short-acting ß$_2$-agonist.
